# Efficacy of Intralesional Triamcinolone Acetonide Combined With Cryotherapy Compared to Intralesional Triamcinolone Acetonide Alone in Treating Limited Patchy Alopecia Areata of the Scalp and Face: A Prospective, Randomized, Within‐Subject Controlled Trial

**DOI:** 10.1111/jocd.70667

**Published:** 2026-02-01

**Authors:** Sama Heidari, Reza Omid, Hanie Babaie, Fatemeh Hosseini, Navid Namakizadeh Esfahani, Zeinab Aryanian

**Affiliations:** ^1^ Autoimmune Bullous Disease Research Center, Razi Hospital Tehran University of Medical Sciences Tehran Iran; ^2^ Department of Dermatology, Razi Hospital Tehran University of Medical Sciences Tehran Iran; ^3^ Department of Epidemiology and Biostatistics, School of Public Health Tehran University of Medical Sciences Tehran Iran

**Keywords:** alopecia areata, combination therapy, cryotherapy, injection, patchy alopecia, triamcinolone

## Abstract

**Background:**

Alopecia areata (AA) is a common autoimmune disease characterized by non‐scarring hair loss. The disease occurs in both sexes, with no racial or age‐related predilection. Many patients experience spontaneous improvement and regrowth of hair, while some patients are resistant to common therapies and others progress to more severe forms of alopecia. The primary treatment for patchy and limited alopecia of the scalp and face is intralesional triamcinolone injection. Previous studies have also reported the efficacy of cryotherapy (liquid nitrogen), which promotes hair regrowth through mechanisms such as local vasodilation and targeting keratinocyte antigens.

**Aims:**

Since both modalities have been used independently, we aimed to evaluate the synergistic effect of both treatments in combination and compare it with triamcinolone injection alone.

**Methods:**

In this prospective, randomized, within‐subject controlled trial, 22 participants with limited patchy alopecia affecting the scalp and face were enrolled. Each patient had symmetrical patches randomly assigned to two treatment modalities: one side received a combination of cryotherapy followed by an intralesional injection of triamcinolone acetonide (8 mg/mL for scalp and 4 mg/mL for face), while the contralateral side received intralesional triamcinolone acetonide alone. Treatments were administered in four consecutive sessions at 4‐week intervals. The primary outcome was the assessment of hair loss severity using the Severity of Alopecia Tool (SALT) score for the scalp and the ALBAS score for the facial area, measured at each visit. Adverse events were evaluated throughout the treatment period.

**Results:**

Both treatment methods demonstrated a statistically significant difference in within‐group analysis (*p* < 0.001). However, a comparison of the two treatments did not reveal any statistically significant superiority between the methods (*p* > 0.05). Additionally, no major adverse effects were observed.

**Conclusion:**

This study demonstrated that both combination therapy and triamcinolone injection are equally effective in treating alopecia. However, future research with a larger sample size, extended follow‐up, and comparisons of trichoscopic findings is warranted.

## 1 Introduction

Alopecia areata (AA) is a prevalent, non‐scarring autoimmune‐based disease, affecting 2% of the worldwide population. It is characterized by well‐defined hair loss patches attributed to the anagen hair follicles, equally affecting both genders, with no age or racial preference [1, 2, 3, 4].

Although the exact pathogenesis of AA remains unclear, immunological dysregulation plays a key role as the underlying cause of disease manifestation. In subsequent stages, trigger factors such as genetic susceptibility, epigenetic modifications, and environmental influences—such as infections—contribute to both the initiation and recurrence of the disease [5, 6].

Changes in physical appearance among patients with AA can lead to a higher prevalence of psychological disorders, including anxiety, depression, and suicidal behavior. The lifetime incidence of psychiatric comorbidities has been reported to be as high as 74%. Therefore, addressing the psychological and emotional well‐being of these patients is a crucial component of treatment [7, 8].

Several therapeutic modalities such as topical treatments (corticosteroids and minoxidil), intralesional (corticosteroids and vitamin D), systemic (methotrexate, cyclosporine, and azathioprine), and phototherapy are used for treating AA. However, the management of AA is still challenging, with high rates of relapse and adverse effects [9, 10]. The primary target of treatment is immunological regression and hair growth stimulation. In adults with confined patches (less than 50% of scalp involvement), intralesional corticosteroids (ILC), specifically triamcinolone acetonide (TA), would be the first‐line treatment option [1, 11, 12].

In recent years, cryotherapy has been introduced as an emerging treatment modality for AA, with varying degrees of clinical success. Its therapeutic effects are thought to be mediated through vascular alterations and immunomodulatory mechanisms [13, 14].

This study aims to evaluate the clinical efficacy of combining ILC (TA) with an unconventional treatment such as cryotherapy, and to compare this combination with intralesional TA monotherapy.

## 2 Materials and Methods

This was a prospective, randomized, within‐subject controlled trial conducted at Dermatology Hospital between June 2023 and June 2024. The study protocol was approved by the Ethics Committee of the Department of Dermatology and the University of Medical Sciences. Written informed consent was obtained from all participants prior to enrollment. The study was conducted in accordance with the principles outlined in the Declaration of Helsinki.

Patients aged more than 15 who were diagnosed with symmetrical, limited patchy lesions of alopecia areata in the scalp and face were included in the study, and exclusion criteria were as follows: patients with extensive patches who are eligible for systemic therapies, patients with systemic and other dermatological diseases, patients with coagulation disorders, patients who have taken treatments for alopecia during the last 3 months, intolerant patients to cold and pain, patients with active secondary infection, individuals who are unwilling to participate in the study, and pregnant and lactating women.

### 2.1 Treatment Allocation and Randomization

Patients' symmetrical patches were randomly assigned to two groups: one treated with cryotherapy in combination with a TA injection, and the other treated with a TA injection alone. Random allocation of the patients was performed using block randomization and random allocation software. A list of random numbers for blocks of 4, 6, and 8 was utilized. If the number chosen was between 0 and 4, the right side of the scalp or face was treated with the combination treatment (cryotherapy plus triamcinolone injection), while the left side was treated with the triamcinolone injection alone. If the selected number was between 5 and 9, the right side of the scalp or face was treated with the triamcinolone injection alone, and the left side was treated with the combination treatment.

### 2.2 Intervention

For each patient, one of the symmetrical alopecia patches was treated with a combination of cryotherapy and ILC injection. Regrading cryotherapy protocol, two passes of a freeze–thaw cycle using liquid nitrogen spray were applied in a brush‐like manner. A thawing period of approximately 2–3 min was allowed to ensure complete tissue thawing.

### 2.3 Intralesional Corticosteroid Injection

Following cryotherapy, the lesion was cleansed with an alcohol pad, and triamcinolone acetonide (40 mg/mL) was diluted with 2% lidocaine to achieve final concentrations of 8 mg/mL for scalp lesions and 4 mg/mL for facial lesions prior to intralesional injection. This solution was intradermally injected at 1 mm intervals using a 31‐gauge syringe. The contralateral patch was treated with triamcinolone injection alone, following the same sterilization and injection protocol but without prior cryotherapy. All patients underwent a total of four treatment sessions, each spaced 4 weeks apart.

### 2.4 Primary Endpoint

The primary endpoint was the evaluation of hair loss severity using the Severity of Alopecia Tool (SALT) score for the scalp and the Alopecia Barbae Severity (ALBAS) score for the facial area [15, 16]. The ALBAS score is a tool analogous to the SALT score and was used to assess each treated side. These scores were calculated at every visit. Standardized photographs were taken at baseline and at each follow‐up visit before the procedures. Hair loss severity was determined by multiplying the percentage of the affected surface area by the percentage of hair loss. These assessments were performed by a dermatologist who was not involved in the treatment procedures to ensure objectivity.

The distribution of percentage surface areas was as follows: *Scalp*: Left side (18%), right side (18%), top (40%), and back (24%); *Face*: Left side (19%), right side (19%), front (12%), and neck area (50%).

### 2.5 Secondary Endpoints

Secondary endpoints included assessing the trend of recovery between the two methods and recording any adverse effects. Additionally, patients were followed for 5 months after treatment completion to evaluate the durability of the treatment effects and the recurrence of lesions.

### 2.6 Statistical Analysis

For descriptive analysis of quantitative data, mean and standard deviation (mean ± SD), median, and the interquartile range (median (IQR)) were used. For qualitative data, frequency and percentage (*N* (%)) were utilized. Additionally, for inferential analysis of data, statistical tests such as the one‐sample Kolmogorov–Smirnov test were applied to check the normality of quantitative data, the reappeared measure ANOVA or its non‐parametric equivalent, the Friedman test, was used to compare quantitative variables during time, and the independent *t*‐test or its non‐parametric equivalent, the Mann–Whitney test, was employed to compare quantitative variables between the Injection and Combination treatments (groups). Furthermore, the R programming language was utilized for data analysis. A significance level of *p* < 0.05 was considered for hypothesis testing.

## 3 Results

A total of 22 participants with limited, patchy AA affecting the scalp and face were enrolled in the study. The mean age of the patients was 30.05 ± 9.7 years, with ages ranging from 15 to 49 years. The mean duration of the disease was 18.09 ± 27.9 months, with a range of 2 to 120 months. The male patient population was predominantly higher than that of females, with 14 cases (63.6%) and 8 cases (36.4%), respectively (Table 1).

**TABLE 1:** demographic data of included patients.

Characteristics		
Age (mean ± SD, range)		30.05 ± 9.7 (15–49 years)
Gender	Female	8 (36.4%)
	Male	14 (63.6%)
Disease duration (mean ± SD, range)		18.09 ± 27.9 (2–120 months)
Location of hair loss	Face	6 (27.3%)
	Scalp	16 (72.7%)
Family history	Yes	5 (22.7%)
	No	17 (77.3%)

The mean hair loss scores for the injection and combination methods were 6.24 ± 3.83 and 7.29 ± 5.11, respectively, prior to any intervention. Following four treatment sessions, the mean hair loss score decreased to 0.83 ± 2.37 in the injection areas and 0.88 ± 2.43 in the combination‐treated areas. Although both approaches showed substantial improvement, a descriptively greater reduction was observed in the combination‐treated areas.

Comparing the efficacy of the two treatments on the scalp and facial areas demonstrated that the injection method resulted in a greater reduction of the hair loss score in the facial area, while the combination treatment led to a more significant decrease in the SALT score for the scalp area (Table 2).

**TABLE 2:** Hair loss (SALT and ALBAS) scores during different visit times based on the location of hair loss and treatment method.

	Group	Location of hair loss	Mean ± SD	Range
Score (Baseline)	Injection	Scalp	5.91 ± 3.33	1.8–11.7
		Face	7.14 ± 5.19	1.95–16.8
	Combination	Scalp	8.77 ± 5.02	3.6–18.9
		Face	3.33 ± 2.81	0.8–7.65
Score (Visit 1)	Injection	Scalp	4.38 ± 3.45	0.4–11.7
		Face	4.64 ± 3.11	1.92–9.6
	Combination	Scalp	6.09 ± 3.79	0.72–13.1
		Face	1.59 ± 1.15	0.65–3.20
Score (Visit 2)	Injection	Scalp	2.31 ± 2.72	0–11.2
		Face	1.53 ± 1.59	0–3.72
	Combination	Scalp	2.58 ± 2.77	0–10.8
		Face	0.66 ± 0.78	0–2
Score (Visit 3)	Injection	Scalp	1.14 ± 2.74	0–10.8
		Face	0 ± 0	0–0
	Combination	Scalp	1.09 ± 2.8	0–10.8
		Face	0.32 ± 0.64	0–1.68

Both injection and combination treatment methods demonstrated a statistically significant decrease in hair loss scores during the follow‐up sessions (*p* < 0.001). Although the improvement was descriptively greater with the combination method, no statistically significant difference was observed between the injection and combination treatments (*p* > 0.05) (Table 3).

**TABLE 3:** Comparison of hair loss score (SALT and ALBAS scores) reduction between the two treatment methods at different follow‐ups.

	Group	Baseline	Time1	Time2	Time3	*p* ^a^
		Mean ± SD	Mean ± SD	Mean ± SD	Mean ± SD	
		Median (IQR)	Median (IQR)	Median (IQR)	Median (IQR)	
SALT and ALBAS scores	Injection	6.24 ± 3.83	4.45 ± 3.29	2.10 ± 2.45	0.83 ± 2.37	< 0.001
		5.85 (5.15)	3.55 (4.54)	1.37 (2.61)	0 (0.4)	
	Combination	7.29 ± 5.11	4.86 ± 3.85	2.06 ± 2.53	0.88 ± 2.43	< 0.001
		6.3 (7.43)	3.4 (6.4)	1.44 (2.44)	0 (0.08)	
	*p* ^b^	0.69	0.78	0.76	0.61	

^a^ Friedman test.

^b^ Mann–Whitney test.

**FIGURE 1:**
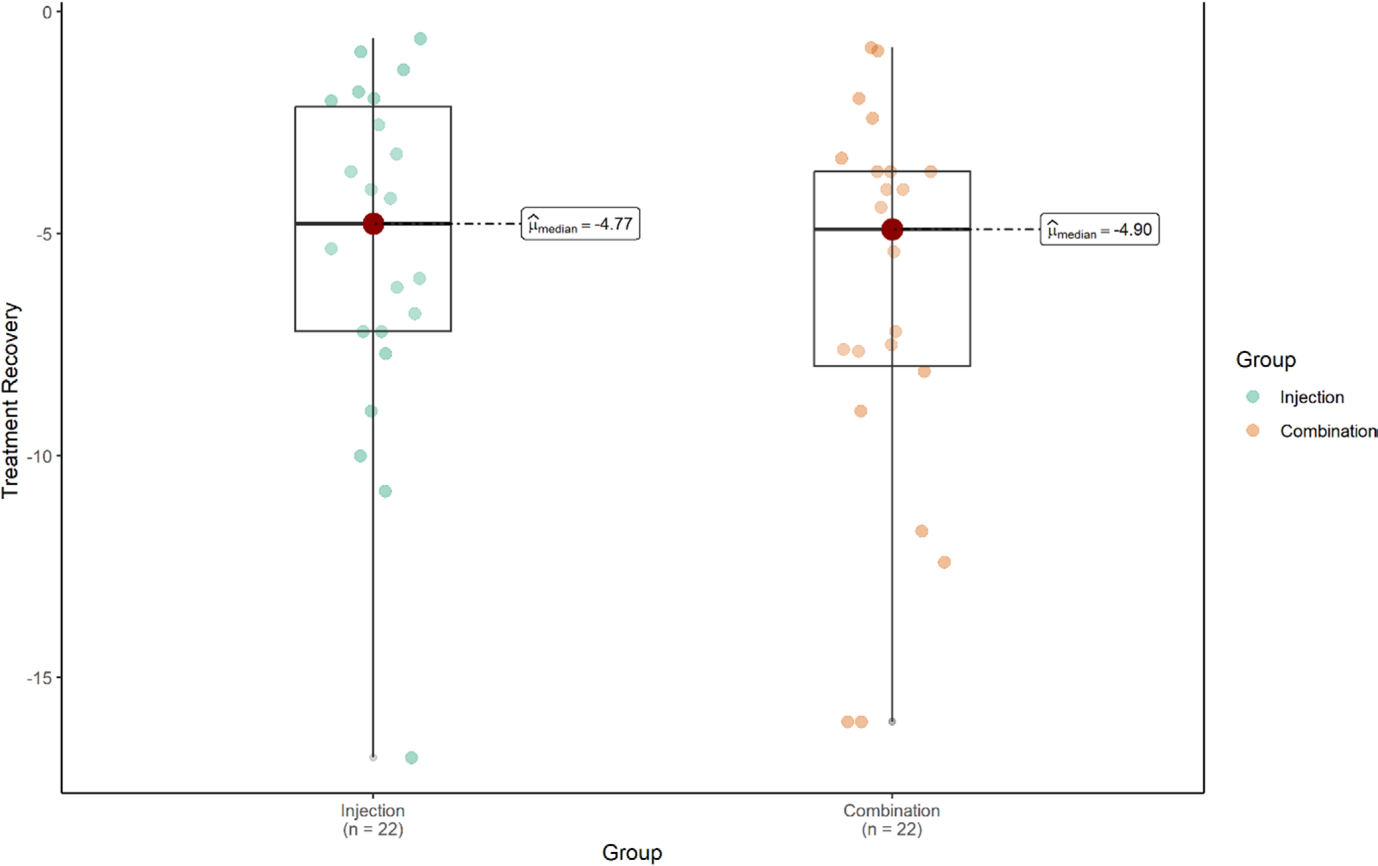
Comparison of trend of recovery between the two treatment methods.

The differences between the treatment methods during the sessions are illustrated in Figure 1, which shows that the trend of recovery was greater in the combination method, although this difference was not statistically significant (*p* = 0.43). Most patients did not experience any adverse effects during the study. Swelling following cryotherapy was reported in one patient (4.5%), while localized atrophy at the injection sites was observed in four patients (18.2%). During the 5‐month follow‐up period after treatment, recurrence of alopecia lesions was noted in seven patients (31.8%), whereas 15 patients (68.2%) showed no signs of recurrence.

**FIGURE 2:**
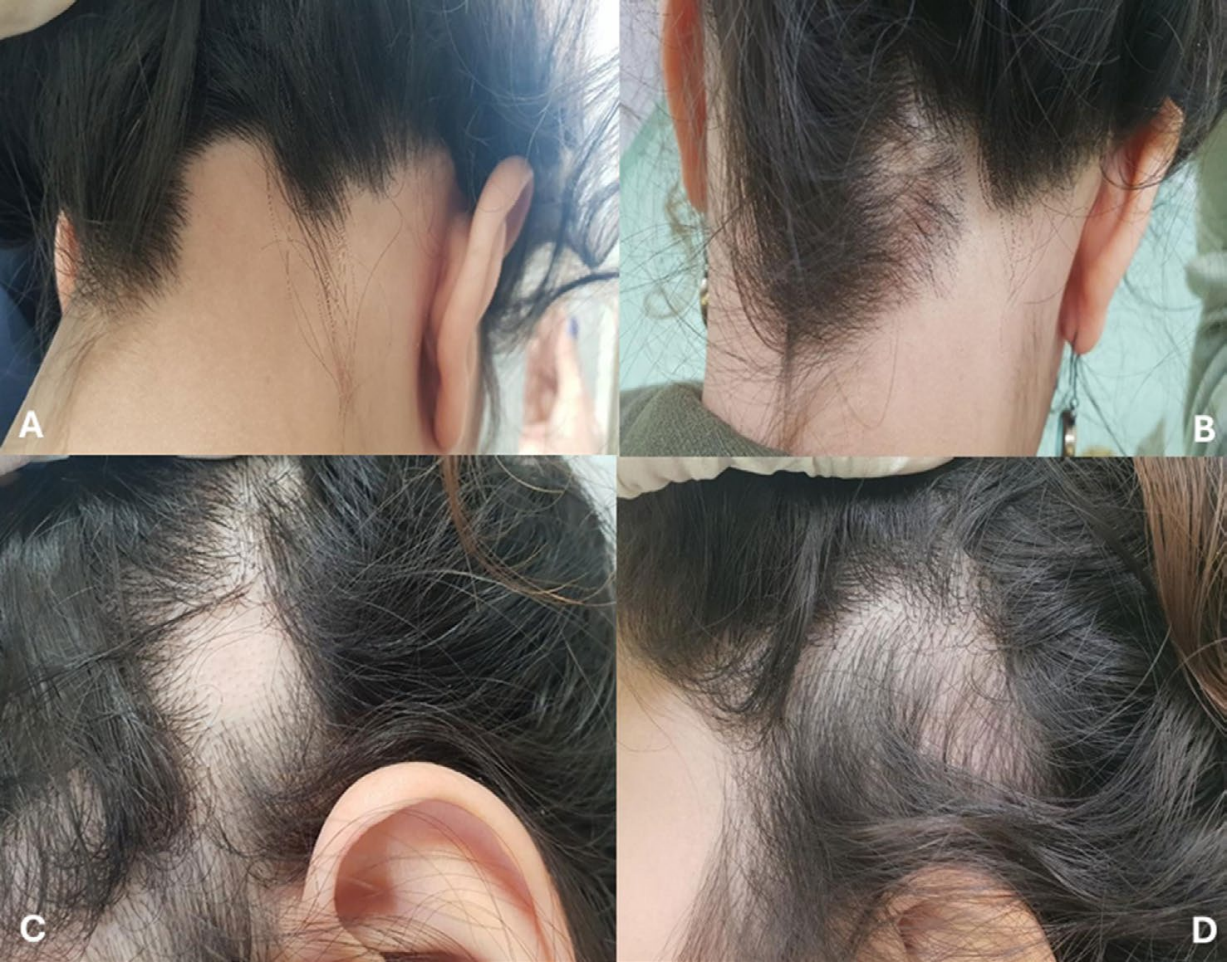
Baseline (A, C) and post‐treatment images at the fourth treatment session (B, D) of a patient with patchy alopecia of the scalp.

**FIGURE 3:**
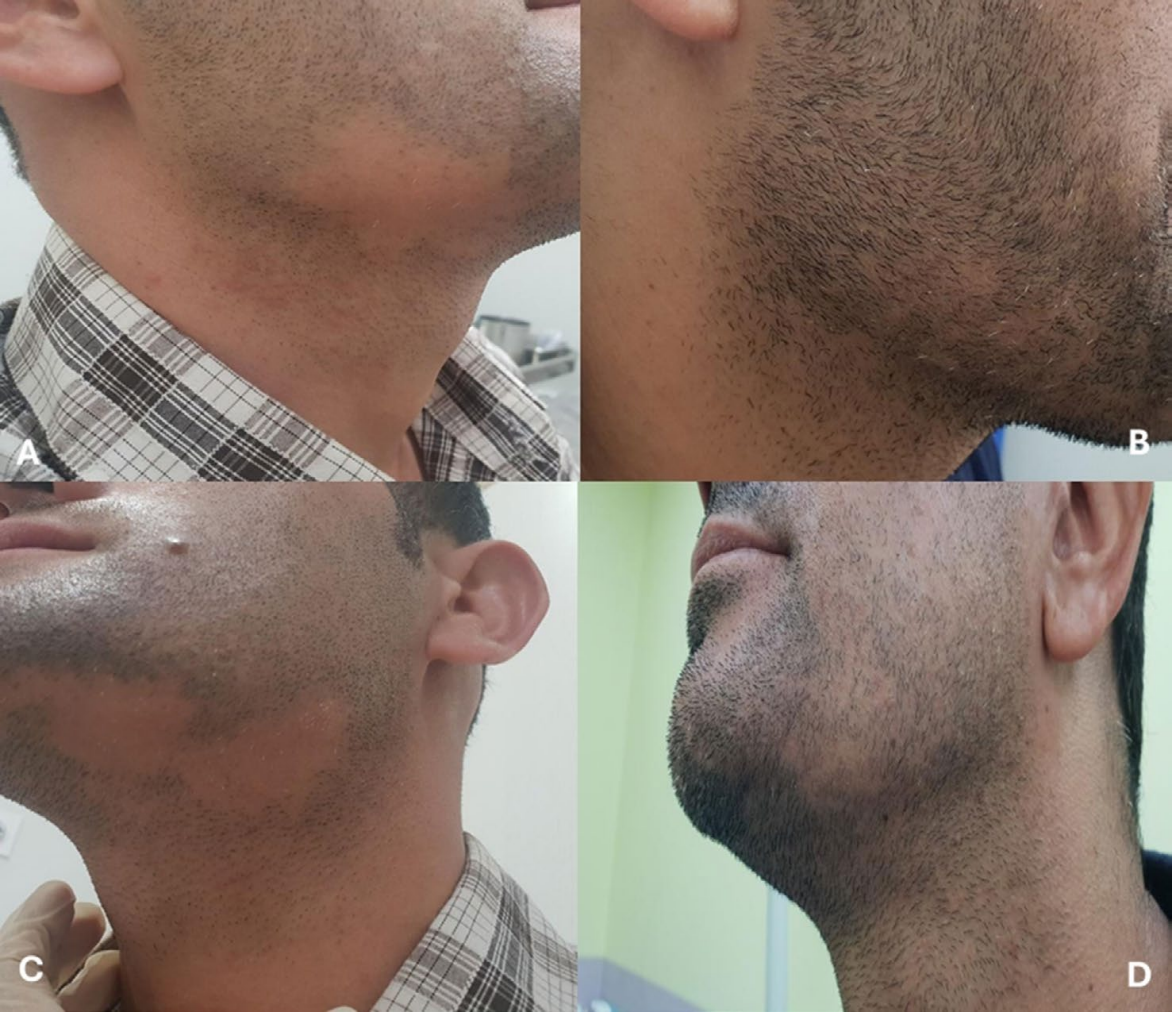
Baseline (A, C) and post‐treatment images at the fourth treatment session (B, D) of a patient with patchy alopecia of the face.

Figures 2 and 3 show clinical images of two patients with scalp and facial alopecia before and after four treatment sessions.

## 4 Discussion

Alopecia areata (AA) is a non‐scarring hair loss disease that is regarded as an organ‐specific autoimmune disease of the hair follicles with a genetic predisposition. Hair follicles are immune‐privileged sites (IPS), which means that they are protected from being distinguished by the immune system. In addition, IPS contain only small amounts of major histocompatibility complex (MHC). When immune privilege breaks down, there is an infiltration of mainly CD8+ and CD4+ T lymphocytes and dendritic cells and, to some extent, natural killer (NK) cells and an increased presence of antigen‐presenting cells such as Langerhans cells. The induced expression of MHC I and II is increased, and cytokines such as IFN‐γ, TNF‐α, IL‐2, and IL‐15 are also released by the immune cells around the hair follicles. This process leads to inflammation and telogen effluvium and/or dystrophic anagen effluvium, resulting in AA [17, 18, 19]. Our study aimed to compare the efficacy of intralesional triamcinolone with that of a combination of intralesional triamcinolone and cryotherapy. Although both modalities demonstrated remarkable outcomes in treating alopecia of both scalp and face, no significant difference was found between them. Additionally, the injection method was more effective on the facial region, while combination therapy showed a greater decrease in SALT score on the scalp area. This incidence may be explained by differences in skin thickness across various body sites. Previous studies have shown that the scalp epidermis tends to thicken during inflammatory processes, which could reduce the efficacy of monotherapy in this area [20]. Moreover, factors such as age, sex, and race also play important roles in determining skin thickness and, consequently, treatment responsiveness [21, 22, 23].

Intralesional injection of corticosteroids such as TA has been an effective treatment for patchy limited alopecia sites; according to a guideline by Messenger et al., the highest preferred efficacy is 5–10 mg/mL for less than 50% scalp involvement in adults. Although a higher dose of corticosteroids is associated with a higher response rate, a greater rate of skin atrophy is possible, as the development of adverse events is dose‐dependent [24, 25].

In this study, we opted to use a relatively high dose of intralesional triamcinolone, 8 mg/mL for scalp alopecia and 4 mg/mL for facial areas. To minimize adverse events, treatment sessions were scheduled at 4‐week intervals. In cases of skin atrophy, subsequent injections were administered at different areas of the lesion using lower doses. We also aimed to evaluate the potential synergistic effect of combining intralesional triamcinolone with cryotherapy, and to compare this combination with intralesional triamcinolone monotherapy, as corticosteroid injections are widely recognized as the most effective first‐line treatment for patchy alopecia [26, 27].

Several studies have assessed intralesional corticosteroids (ILC) as monotherapy compared with cryotherapy; however, to our knowledge, no study has yet examined the efficacy of combining ILC with cryotherapy. The results of existing studies comparing ILC and cryotherapy have been inconsistent. Two studies found ILC to be significantly more effective than cryotherapy [28, 29], whereas a study by Mohammed et al. reported no significant difference between the two treatments in terms of SALT score and hair regrowth [30]. Interestingly, a comparative study by El‐Sayed et al. showed cryotherapy to be significantly more effective than ILC (*p* = 0.002) [31]. These discrepancies may be attributed to variations in cryotherapy technique and timing, treatment intervals, patient age, and lesion size [32].

In a systematic review by Lim et al., 64% of alopecia areata (AA) patients treated with cryotherapy demonstrated hair regrowth exceeding 25%. Moreover, studies that employed combination therapies involving cryotherapy reported enhanced treatment efficacy [33].

In a recently published pilot study by Lee and colleagues, 15 patients were enrolled. An alopecic patch of each patient was randomly divided into four quadrants: negative control, two cycles of 10 s of cryotherapy, two cycles of 20 s of cryotherapy, and intralesional triamcinolone (2.5 mg/mL). Patients underwent six treatment sessions at 2‐week intervals, and clinical response was evaluated based on hair regrowth percentage. Compared to negative controls, treatment with triamcinolone and 20 s of cryotherapy indicated a statistically significant difference in terms of hair regrowth (*p* = 0.038 and *p* = 0.048, respectively). However, 10 s of cryotherapy did not show any improvement in hair regrowth (*p* = 0.97) [34]. In this regard, the first study that compared different durations of cryotherapy on AA patients showed that the mean percentage of improvement was significantly higher in two groups of patients with 8–10 and 13–15 s of cryotherapy compared to the group with 3–5 s [35]. These studies revealed the importance of the duration of cryotherapy conduction on alopecic patches, which might exhibit a greater clinical response.

Cryotherapy plays a significant role in promoting hair regrowth in AA through several proposed mechanisms. Initially, cryotherapy induces local vasoconstriction followed by vasodilation of surrounding blood vessels, leading to increased blood flow to the hair follicles. Additionally, exposure to cold triggers a sympathetic nervous system response, which promotes activation of hair follicle stem cells (HFSCs), thereby stimulating hair regrowth. Cryotherapy also causes destruction of Langerhans cells and keratinocytes; by damaging the antigenic components of these cells, it reduces the autoimmune attack on hair follicles and facilitates regrowth [36, 37, 38, 39].

ILC promote hair regrowth primarily by inhibiting the T‐cell–mediated immune response responsible for attacking hair follicles. However, ILC treatment can be associated with adverse effects, including injection site pain, skin and follicular atrophy, telangiectasia, and pigmentary changes such as hypopigmentation or depigmentation. Among these, pain and transient skin atrophy are the most frequently reported side effects, as also observed in our patient cohort [40, 41].

The limitations of our study include a small sample size and a relatively short follow‐up duration. Additionally, we were unable to assess hair regrowth using trichoscopy, which could have provided further clinical insight and strengthened the study's findings. Finally, patients were not categorized according to the clinical course of AA (active, long‐standing, or biphasic), which should be considered in future studies.

## 5 Conclusion

Although the combination group showed a greater descriptive improvement in hair loss scores, both treatment modalities demonstrated encouraging effectiveness in promoting hair regrowth. Future studies with larger sample sizes, extended follow‐up periods, and the inclusion of trichoscopic assessment are recommended to validate and expand upon these findings.

## Author Contributions

Conceptualization: Z.A.; methodology: Z.A, S.H.; supervision: Z.A., data curation: S.H., R.O.; writing‐original draft: S.H., R.O.; writing‐review and editing: S.H., H.B., N.N.E.; data analyzing: F.H.

## Funding

The study was funded by Tehran University of Medical Sciences. This research received no specific grant number.

## Ethics Statement

The study has been approved by the ethics committee of Razi Hospital and Tehran University of Medical Sciences, IR.TUMS.MEDICINE.REC.1400.1276, IRCT number: IRCT20230124057201N1.

## Conflicts of Interest

The authors declare no conflicts of interest.

## Data Availability

The data analyzed in this study are not publicly available due to privacy considerations but may be obtained from the corresponding author upon reasonable request.

## References

[1] A. Balakrishnan , B. Joy , A. Thyvalappil , P. Mathew , A. Sreenivasan , and R. Sridharan , “A Comparative Study of Therapeutic Response to Intralesional Injections of Platelet‐Rich Plasma Versus Triamcinolone Acetonide in Alopecia Areata,” Indian Dermatology Online Journal 11 (2020): 920–924. 33344340 10.4103/idoj.IDOJ_6_20 PMC7734998

[2] H. Prasanna , R. T. Srinivas , S. K. Kuppuswamy , H. Keloji , and P. T. Ravikumar , “A Comparative Study of Fractional CO_2_ Laser With Topical Triamcinolone Acetonide Versus Intralesional Triamcinolone Acetonide in the Treatment of Alopecia Areata,” Journal of Cutaneous and Aesthetic Surgery 17 (2024): 34–40, 10.4103/JCAS.JCAS_31_23. 38736853 PMC11086937

[3] B. E. Yee , Y. Tong , A. Goldenberg , and T. Hata , “Efficacy of Different Concentrations of Intralesional Triamcinolone Acetonide for Alopecia Areata: A Systematic Review and Meta‐Analysis,” Journal of the American Academy of Dermatology 82 (2020): 1018–1021, 10.1016/j.jaad.2019.11.066. 31843657

[4] A. Hussain , S. A. A. Gardezi , S. Ilyas , N. Sultan , and S. Aman , “Comparison of Efficacy of Intra‐Lesional Injections of Platelet Rich Plasma Therapy Versus Intralesional Triamcinolone Acetonide in the Treatment of Patients With Alopecia Areata at a Tertiary Care Hospital,” Journal of Pakistan Association of Dermatologists 33 (2023): 637–642.

[5] K. Wojciechowska , M. Brzozowska , W. Wardal , et al., “Alopecia Areata: Pathogenesis, Current Treatments, and Future Perspectives,” Journal of Education, Health and Sport 79 (2025): 58241.

[6] T. Ma , T. Zhang , F. Miao , et al., “Alopecia Areata: Pathogenesis, Diagnosis, and Therapies,” MedComm 6 (2025): 1–30, 10.1002/mco2.70182. PMC12010142 40260013

[7] M. Devi , A. Rashid , and R. Ghafoor , “Intralesional Triamcinolone Acetonide Versus Topical Betamethasone Valearate in the Management of Localized Alopecia Areata,” Journal of the College of Physicians and Surgeons–Pakistan 25 (2015): 860–862. 26691357

[8] R. Qiao , J. Zhu , J. Fang , et al., “Microneedle Transdermal Delivery of Compound Betamethasone in Alopecia Areata—A Randomized Controlled Trial,” Journal of the American Academy of Dermatology 92 (2024): 269–275, 10.1016/j.jaad.2024.09.059. 39393548

[9] H. M. Abdo , E. M. Elrewiny , A. Shawky , A. M. Ammar , A. M. Atef , and M. A. Rageh , “Intralesional Pentoxifylline Injection Versus Triamcinolone Acetonide in Treating Localized Alopecia Areata: A Comparative Study,” Journal of Clinical and Aesthetic Dermatology 16 (2023): 26–30. PMC10703504 38076656

[10] M. H. Ragaie , S. E. Mohammed , and S. S. Shehata , “Intralesional Vitamin D3 Versus Intralesional Triamcinolone Acetonoid in Patchy Alopecia Areata: A Comparative Clinical and Dermoscopic Study,” Journal of Cosmetic and Laser Therapy 00 (2024): 1–5, 10.1080/14764172.2024.2389275. 39139085

[11] T. W. Chu , M. Aljasser , A. Alharbi , O. Abahussein , K. McElwee , and J. Shapiro , “Benefit of Different Concentrations of Intralesional Triamcinolone Acetonide in Alopecia Areata: An Intrasubject Pilot Study,” Journal of the American Academy of Dermatology 73 (2015): 338–340, 10.1016/j.jaad.2015.04.049. 26183987

[12] A. Hamza , A. Elsayed , and A. Abdel‐Bary , “Combined Intralesional Platelet‐Rich Plasma and Intralesional Steroid Versus Intralesional Steroid Alone in the Treatment of Alopecia Areata,” Journal of the Egyptian Women's Dermatologic Society 20 (2023): 98–105, 10.4103/jewd.jewd_53_22.

[13] M. Jun and W. S. Lee , “Therapeutic Effect of Superficial Cryotherapy on Alopecia Areata: A Prospective, Split‐Scalp Study in Patients With Multiple Alopecia Patches,” Annals of Dermatology 29 (2017): 722–727, 10.5021/ad.2017.29.6.722. 29200760 PMC5705353

[14] D. S. Sayed , A. A. Allam , and E. M. Abdel‐Majid , “Superficial Cryotherapy Versus Topical Psoralen and Ultraviolet A in the Treatment of Alopecia Areata: A Randomized, Controlled Trial,” Journal of the Egyptian Women's Dermatologic Society 17 (2020): 98–103, 10.4103/JEWD.JEWD_7_20.

[15] E. Olsen , M. Hordinsky , S. McDonald‐Hull , et al., “Alopecia Areata Investigational Assessment Guidelines,” Journal of the American Academy of Dermatology 40 (1999): 242–246, 10.1016/S0190-9622(99)70195-7. 10025752

[16] A. J. Stefanis , P. Arenberger , M. Arenbergerova , and S. Gkalpakiotis , “Alopecia Barbae Severity Score: A Novel Scoring System to Estimate the Extent of Beard Loss and Success of Treatment,” British Journal of Dermatology 185 (2021): 847–849, 10.1111/bjd.20489. 33997953

[17] R. M. Trüeb and M. F. R. G. Dias , “Alopecia Areata: A Comprehensive Review of Pathogenesis and Management,” Clinical Reviews in Allergy and Immunology 54 (2018): 68–87, 10.1007/s12016-017-8620-9. 28717940

[18] F. Rajabi , L. A. Drake , M. M. Senna , and N. Rezaei , “Alopecia Areata: A Review of Disease Pathogenesis,” British Journal of Dermatology 179 (2018): 1033–1048, 10.1111/bjd.16808. 29791718

[19] L. C. Strazzulla , E. H. C. Wang , L. Avila , et al., “Alopecia Areata: Disease Characteristics, Clinical Evaluation, and New Perspectives on Pathogenesis,” Journal of the American Academy of Dermatology 78 (2018): 1–12, 10.1016/j.jaad.2017.04.1141. 29241771

[20] C. Ekelem , N. Feil , B. S. E. Csuka , et al., “Optical Coherence Tomography in the Evaluation of the Scalp and Hair: Common Features and Clinical Utility,” Lasers in Surgery and Medicine 53, no. 1 (2021): 129–140, 10.1002/lsm.23243. 32253781

[21] A. Firooz , H. Zartab , N. Pazhohi , F. Fanian , and L. Janani , “The Influence of Gender and Age on the Thickness and Echo‐Density of Skin,” Skin Research and Technology 23, no. 1 (2016): 13–20, 10.1111/srt.12294. 27273751

[22] D. A. Lintzeri , “Epidermal Thickness in Healthy Humans: A Systematic Review and Meta‐Analysis,” Journal of the European Academy of Dermatology and Venereology 36 (2022): 1191–1200, 10.1111/jdv.18123. 35366353

[23] C. Cakmakoglu , G. Kwiecien , L. N. Raborn , and J. R. Gatherwright , “The Influence of Age and Gender on Forehead Soft Tissue Thickness: A Magnetic Resonance Imaging‐Based Study,” Aesthetic Plastic Surgery 49 (2025): 5275–5283, 10.1007/s00266-025-04957-y. 40464860 PMC12594695

[24] A. G. Messenger , J. McKillop , P. Farrant , A. J. McDonagh , and M. Sladden , “British Association of Dermatologists' Guidelines for the Management of Alopecia Areata 2012,” British Journal of Dermatology 166 (2012): 916–926, 10.1111/j.1365-2133.2012.10955.x. 22524397

[25] H. A. Su , Y. T. Chen , and Y. C. Chen , “The Optimal Concentration of Intralesional Triamcinolone Acetonide for Patchy Alopecia Areata: A Systematic Review and Meta‐Analysis,” Dermatologica Sinica 40 (2022): 85–93, 10.4103/ds.ds_15_22.

[26] S. Awasthi , M. Nijhawan , A. Mishra , and A. Gupta , “Comparing the Efficacy of Oral Apremilast, Intralesional Corticosteroids, and Their Combination in Patients With Patchy Alopecia Areata: A Randomized Clinical Controlled Trial,” Archives of Dermatological Research 317 (2025): 3–9, 10.1007/s00403-024-03642-5. 39673617

[27] C. H. Pratt , L. E. King , A. G. Messenger , A. M. Christiano , and J. P. Sundberg , “Alopecia Areata,” Nature Reviews Disease Primers 3 (2017): 1–17. 10.1038/nrdp.2017.11 PMC5573125 28300084

[28] M. Amirnia , S.‐S. Mahmoudi , F. Karkon‐Shayan , et al., “Comparative Study of Intralesional Steroid Injection and Cryotherapy in Alopecia Areata,” Nigerian Medical Journal 56 (2015): 249–252, 10.4103/0300-1652.165034. 26759508 PMC4697211

[29] S. Sardana , T. Goyal , P. Kushwaha , and P. Jha , “A Prospective Study to Compare the Efficacy of Cryotherapy Versus Intralesional Steroid in Alopecia Areata,” Journal of Cutaneous and Aesthetic Surgery 15 (2022): 175–178, 10.4103/JCAS.JCAS_166_20. 35965906 PMC9364455

[30] A. I. A. Mohammed , A. S. Farag , and H. S. A. Hafiz , “Superficial Cryotherapy Versus Intralesional Corticosteroid Injection in Alopecia Areata: A Comparative Clinical and Dermoscopic Study,” Egyptian Journal of Hospital Medicine 86 (2022): 211–217.

[31] M. H. El Sayed , N. E. Ibrahim , and A. A. Afify , “Superficial Cryotherapy Versus Intralesional Corticosteroids Injection in Alopecia Areata: A Trichoscopic Comparative Study,” International Journal of Trichology 14 (2022): 8–13. 35300102 10.4103/ijt.ijt_130_20 PMC8923143

[32] E. Abdel‐Majid , D. S. Abdel‐Kader , and A. Allam , “Liquid Nitrogen Cryotherapy in the Treatment of Alopecia Areata: An Egyptian Study,” Journal of Current Medical Research and Practice 3 (2018): 187, 10.4103/jcmrp.jcmrp_103_18.

[33] S. H. Lim and W. S. Lee , “Hair Regrowth Outcomes of Superficial Cryotherapy in Patients With Alopecia Areata: A Systematic Review,” Annals of Dermatology 35 (2023): 464–467, 10.5021/AD.22.095. 38086361 PMC10733080

[34] H. Lee , J. W. Lee , S. Park , H. Park , G. H. Kim , and O. Kwon , “Comparative Analysis of Temperature‐Controlled Cryotherapy Versus Intralesional Triamcinolone Acetonide Injection for Alopecia Areata: An Intrasubject Split‐Lesion Pilot Study,” Journal of the European Academy of Dermatology and Venereology 1 (2024): 1–3, 10.1111/jdv.20464. 39607009

[35] A. A. Abdel Motaleb and D. S. Sayed , “Different Freezing Time of Superficial Liquid Nitrogen Cryotherapy in Treatment of Recalcitrant Alopecia Areata: Randomized Clinical Trial,” Dermatologic Therapy 33 (2020): e13640, 10.1111/dth.13640. 32441386

[36] Y. Shwartz , M. Gonzalez‐Celeiro , C. L. Chen , et al., “Cell Types Promoting Goosebumps Form a Niche to Regulate Hair Follicle Stem Cells,” Cell 182 (2020): 578–593.e19, 10.1016/j.cell.2020.06.031. 32679029 PMC7540726

[37] S. Lee , S. Kim , S. T. Hwang , G. H. Kim , and O. Kwon , “Cold Shock Therapy Promotes Hair Growth in Association With Upregulation of Cold‐Inducible RNA‐Binding Protein and Vascular Endothelial Growth Factor,” Journal of Dermatological Science 115 (2024): 141–144, 10.1016/j.jdermsci.2024.08.001. 39181732

[38] V. P. Zawar and G. M. Karad , “Liquid Nitrogen Cryotherapy in Recalcitrant Alopecia Areata: A Study of 11 Patients,” International Journal of Trichology 8 (2016): 15–20, 10.4103/0974-7753.179403. 27127370 PMC4830166

[39] M. Radmanesh and M. Azar‐Beig , “Cryotherapy as an Alternative Therapy for the Treatment of Recalcitrant Alopecia Areata,” Iranian Journal of Dermatology 16 (2013): 49–52.

[40] W. Albadri and A. C. Inamadar , “Comparison of Clinical Efficacy and Trichoscopic Changes in Alopecia Areata of the Scalp Following Treatment With Intralesional Triamcinolone Acetonide and Autologous Platelet‐Rich Plasma,” Clinical Dermatology Review 7 (2023): 229–233, 10.4103/cdr.cdr_14_22.

[41] M. Kumaresan , “Intralesional Steroids for Alopecia Areata,” International Journal of Trichology 2 (2010): 63–65, 10.4103/0974-7753.66920. 21188031 PMC3002419

